# Cannabinoids and orthopedic surgery: a systematic review of therapeutic studies

**DOI:** 10.1186/s13018-021-02205-y

**Published:** 2021-01-14

**Authors:** Bradley J. Vivace, Allyson N. Sanders, Steven D. Glassman, Leah Y. Carreon, Joseph L. Laratta, Jeffrey L. Gum

**Affiliations:** 1 University of Louisville School of Medicine, 500 South Preston Street, Louisville, KY USA; 2 Norton Leatherman Spine Center, 210 E Gray Street, Suite 900, Louisville, KY USA

**Keywords:** Cannabinoids, Orthopedic surgery, Analgesia, Opioids, Arthroplasty, Multimodal pain control

## Abstract

**Background:**

Recent work has shed light on the potential benefits of cannabinoids for multimodal pain control following orthopedic procedures. The objective of this review was to summarize the available evidence of analgesic and opioid-sparing effects cannabinoids have in orthopedic surgery and identify adverse events associated with their use.

**Methods:**

A systematic review of the literature using Preferred Reporting Items for Systematic Reviews and Meta-Analyses (PRISMA) guidelines including PubMed, EMBASE, MEDLINE, PsycINFO, and Google Scholar was performed to include all primary, therapeutic studies published on the use of cannabis, and cannabis-derived products in orthopedic surgery.

**Results:**

The literature review returned 4292 citations. Thirteen publications were found to meet inclusion criteria. Four randomized controlled trials were evaluated while the remaining studies were of quasi-experimental design.

**Conclusion:**

Research on cannabinoids in orthopedic surgery is mostly of a quasi-experimental nature and is mainly derived from studies where orthopedics was not the primary focus. The overall results demonstrate potential usefulness of cannabinoids as adjunctive analgesics and in mitigating opioid use. However, the current evidence is far from convincing. There is a need to produce rigorous evidence with well-designed randomized controlled trials specific to orthopedic surgery to further establish these effects.

## Background

Cannabinoids represent an area of emerging research amidst legislative change [1]. A cursory search for “medical cannabis” reveals over half of the 9057 citations indexed in the United States (U.S.) National Library of Medicine are from the last 5 years [2]. Cannabinoids are approved by the U.S. Food and Drug Administration (FDA) for recalcitrant chemotherapy-associated nausea and vomiting [3], acquired immunodeficiency syndrome-related anorexia [4], and certain forms of epilepsy [5]. Outside of the USA, an oral solution of delta-9-tetrahydrocannabinol (THC) and cannabidiol (CBD) is indicated for refractory multiple sclerosis-associated spasticity [6]. The use of cannabinoids has been investigated in other applications including chronic pain, appetite stimulation, glaucoma, and anxiety [7]. The effects of these compounds have been studied to a lesser extent in orthopedic surgery [8].

Madden et al. covered the use of cannabinoids within orthopedic surgery in their 2018 systematic review appraising the literature through 2017 [8]. Since this time, there has been evidence attesting to the benefit of cannabinoids on post-operative recovery and reduction in morphine use following orthopedic procedures [9]. Given the current trend of widespread opioid misuse within the USA [10], this potential to limit narcotic use is promising. Legality has continued to expand [1] and the popularity of cannabinoids has increased where 14% of Americans in 2019 used CBD products, most commonly for pain [11]. The objective of this review was to summarize the available evidence of analgesic and opioid-sparing effects cannabinoids have within orthopedic surgery and identify adverse events (AE) associated with their use.

## Methods

Guidelines from Preferred Reporting Items for Systematic Reviews and Meta-Analyses Protocols (PRISMA) and PRISMA checklist of recommended items to include in a systematic review were utilized in the construction of this systematic review [12] (Additional file 1: PRISMA Checklist).

### Search strategy

**Fig. 1:**
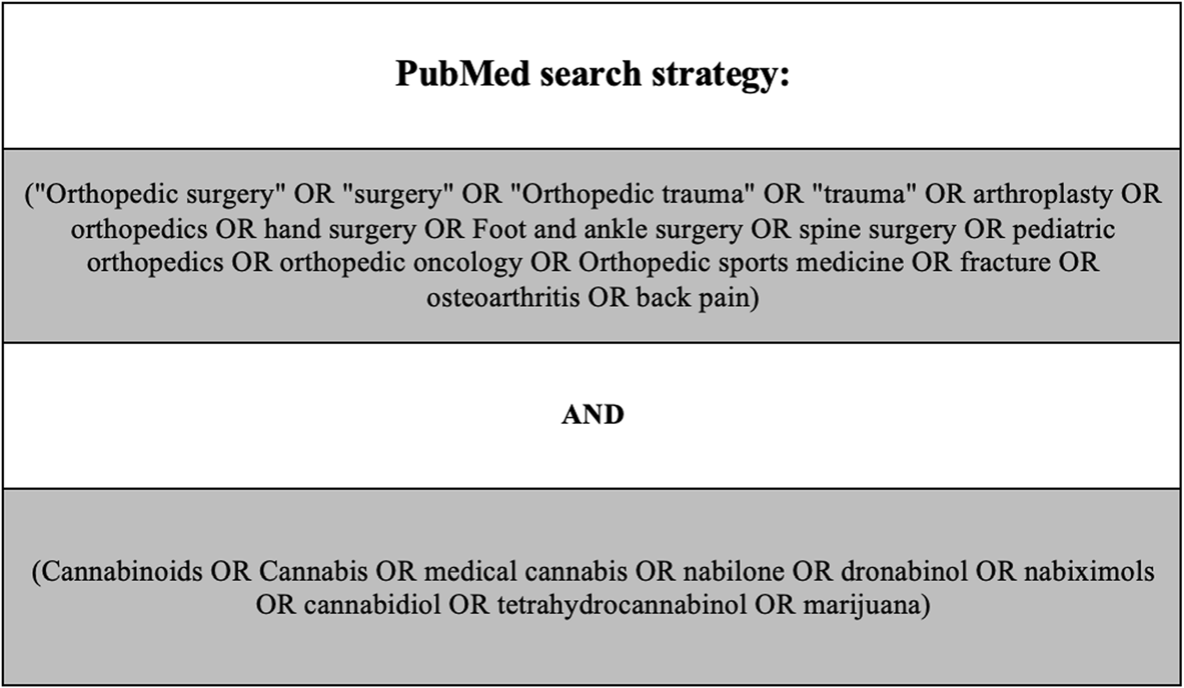
Search terms for the PubMed database. These are the search terms that were used to perform the query of the PubMed database

On March 1st, 2020, the databases EMBASE, PubMed, Ovid MEDLINE, PsycInfo, and Google Scholar were queried. Search terms for orthopedic surgery included “orthopaedic surgery,” “spine surgery,” “orthopaedic procedures,” “osteoarthritis,” “musculoskeletal disease,” and “arthroplasty.” These terms were combined with search terms for cannabinoids that included “cannabinoids,” “medical cannabis,” “cannabis,” “tetrahydrocannabinol,” and “cannabidiol.” Search terms for the PubMed database can be seen in Fig. 1. Other databases were queried in similar fashion (Fig. 1)

### Eligibility criteria

Only primary works of interventional design were considered. Articles combining the therapeutic use of cannabinoids and any subspecialty of orthopedic surgery or conditions common to the field such as fractures, osteoarthritis, and back pain were included. Case reports were excluded, as were non-English and preclinical studies.

### Study selection

A title review was performed of all citations found in each database independently. Duplicate citations were removed, and the abstracts were reviewed. The remaining studies underwent full text review, where each was read individually by at least two of the authors. The review process was performed by BJV and ANS. All disputes of inclusion were settled with the senior authors.

### Data extraction and synthesis

The following data were gathered: study design, population, intervention, control, analgesic effect, differences in opioid use, and AEs. Management of data was done with an electronic chart in which all extracted data was listed by study and utilized for the writing of the manuscript.

### Appraisal of evidence

Evidence of each study was evaluated utilizing the Grading of Recommendations of Assessment, Development, and Evaluation (GRADE) criteria [13] and therapeutic level of evidence was assigned as outlined in the Journal of Bone and Joint Surgery [14]. Bias was assessed with the Revised Cochrane risk-of-bias tool for randomized trials (RoB 2) in randomized controlled trials (RCTs); crossover trials were evaluated with the RoB2 tool specific for crossover trials [15]. The Risk of Bias In Non-Randomized Studies–of Interventions (ROBINS-I) [16] tool was used to assess bias in comparative non-randomized studies. Given the inherent biases found with case series designs, studies without comparative groups were not assessed for bias. Bias was assessed contemporaneously by BJV and ANS.

## Results

**Fig. 2:**
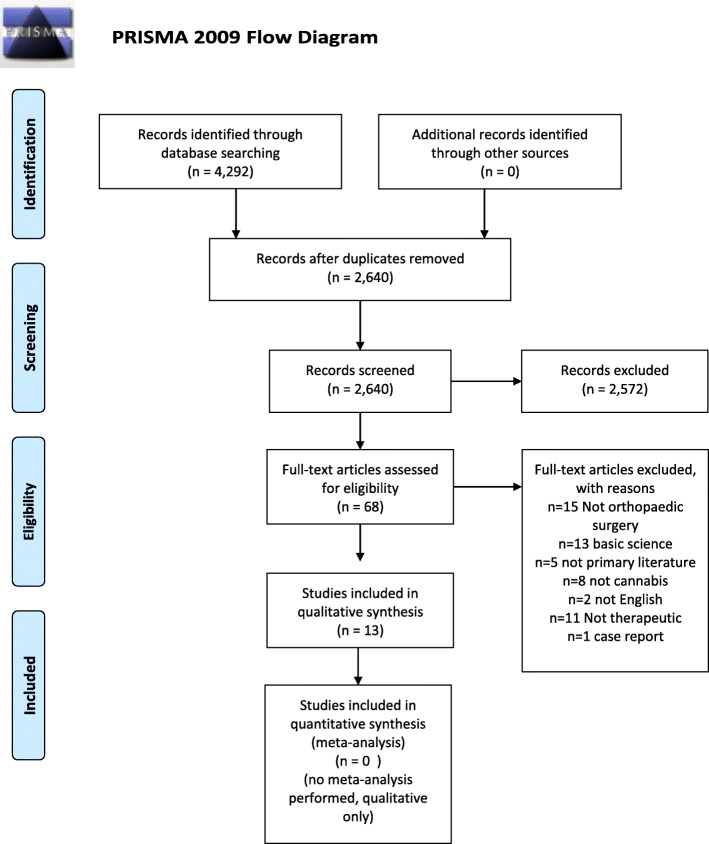
PRISMA flow diagram presenting the systematic review process used in this study. This flow diagram tabulates the process of how the references utilized in this study were obtained

The search produced a total of 4292 titles, and 13 publications met our inclusion criteria. The process in which these 13 references were obtained is tabulated in the PRISMA flowsheet depicted by Moher and colleagues [12] (Fig. 2).

### Design

Of the 13 studies, four were RCTs [17–20]. Nine were quasi-experimental in design. This included two of cohorts [9, 21], three crossovers [22–24], one dose escalation trial [25], and three case series [26–28]. One cohort was retrospective [9] and the other was prospective [21]. Two crossovers were open label [23, 24], while one blinded and randomized the interventions and placebo, but only enrolled subjects in the crossover portion if they benefited during an anteceding, open label two week run-in with THC/CBD [22].

### Appraisal of evidence

No studies were given a high level of evidence via GRADE. Five were evaluated as moderate evidence [17–20, 25], three as low evidence [9, 21, 22], and five as very low [22–24, 26–28]. Regarding therapeutic level of evidence, four provided level I evidence [17–20], two level II evidence [21, 25], one level III evidence [9], and six studies provided level IV evidence [22–24, 26–28] (Table 1). Among RCTs, one was found to have a high risk of bias due to concerns of the randomization process yielding disparate comparative groups [17], two of some concern for bias [18, 19], and one of low risk of bias [20]. Across crossover studies, some concern of bias was found in one [22], while both studies completed by Yassin were judged to be of high risk of bias given their open-label, non-randomized design [23, 24]. All three non-randomized comparative trials were found to be of moderate risk of bias [9, 21, 25] (Table 2).

**Table 1:** Overview and critical appraisal of the identified studies

Study	Beaulieu [17]	Haroutounian et al. [26]	Hickernell et al. [9]	Holdcroft et al. [25]	Hunter et al.^a^ [18]	Jain et al. [19]	Levin et al. [20]	Mondello et al. [27]	Notcutt et al. [22]	Poli et al. [28]	Ware et al. [21]	Yassin et al. [23]	Yassin et al. [24]
Year	2006	2016	2018	2006	2018	1981	2017	2018	2004	2016	2005	2016	2019
Design	RCT	Case series	Retrospective cohort	Dose escalation	RCT	RCT	RCT	Case series	Cross over	Case series	Prospective cohort	Cross over	Cross over
TLE	I	IV	III	II	I	I	I	IV	IV	IV	II	IV	IV
Quality of evidence	M	VL	L	M	M	M	M	VL	L	LV	L	VL	VL
Analgesic effect	−	+	+	+	+^b^	+	0	+	+	+	+	+	+
Opioid use	No change	Decrease	Decrease	Decrease	X	X	No change	X	X	X	X	Decrease	Decrease

This chart contains identified studies, the date of publication, overall assessment of efficacy regarding analgesic effect and change in opioid use, therapeutic level of evidence was assigned as outlined by *JBJS* and quality was assigned by GRADE. (+) a positive effect was noted, (0) a null effect was noted, (−) a negative effect was noted, (X) opioid consumption not examined in study

^a^Abstract only

^b^Conditionally effective

**Table 2:** Risk of bias of included randomized and non-randomized controlled trials

Randomized controlled trials								
Study	Randomization	Deviation from intended effects	Missing outcome data	Measurement of outcome	Selection of the reported result	Overall		
Beaulieu et al. [17]	High	SC	Low	Low	SC	High		
Hunter et al.^a^ [18]	SC	SC	SC	Low	SC	SC		
Jain et al. [19]	Low	Low	Low	Low	SC	SC		
Levin et al. [20]	Low	Low	Low	Low	Low	Low		
Crossover trials								
Study	Randomization	Carry-over	Deviation from intended intervention	Missing outcome data	Measurement of outcome	Selection of reported results	Overall	
Notcutt et al. [22]	Low	SC	Low	Low	Low	Low	SC	
Yassin et al. [23]	High	SC	SC	Low	SC	Low	High	
Yassin et al. [24]	High	SC	SC	Low	SC	Low	High	
Comparative parallel non-randomized trials								
Study	Confounding	Selection	Classification	Deviation from intended intervention	Missing Data	Measurement of outcomes	Selection of reported results	Overall
Hickernell et al. [9]	Moderate	Low	Low	Moderate	Low	Moderate	Low	Moderate
Holdcroft et al. [25]	Moderate	Low	Low	Low	Low	Moderate	Low	Moderate
Ware et al. [21]	Moderate	Moderate	Low	Low	Low	Low	Low	Moderate

This table contains the bias assessment of included randomized controlled trials and comparative non-randomized studies. The headings are individual domains outlined in the RoB 2 and ROBINS-I tools

^a^Abstract only

### Population

Five studies were in the acute perioperative setting [9, 17, 19, 20, 25], including two post THA or TKA [9, 17], one in fracture care [19], and two unspecified orthopedic procedures [20, 25]. The remaining eight studies involved patients with chronic pain from arthritis [18, 21, 22, 28], low back pain [23, 24, 26, 27], and chronic post-surgical pain [22, 27]. Jain et al. included > 90% of male subjects [19], while a female preponderance of greater than 75% of subjects was noted in four studies [17, 20, 24, 25], with one exclusively female [20].

### Intervention

A multiplicity of interventions was used. Oral caplets of cannabinoids were the most common, comprising four studies [9, 17, 20, 25]. Two exclusively utilized the combustion or vaporization of cannabis [23, 24]. Liquid oral administration either sublingual or THC/CBD suspension was used in three studies [22, 27, 28]. One utilized transdermal CBD gel [18], while another used intramuscular levonantradol [19]. Two allowed for patient choice of administration resulting in mixed delivery; most commonly smoked cannabis [21, 26]. The dose, frequency, and potency of cannabinoid varied widely and can be seen by study in Table 3.

**Table 3:** Population, cannabinoid intervention, and control group of identified studies

Study	Population	Intervention	Control
Beaulieu [17]	Adults with post-operative PCA device	*n* = 20 (85% female) Nabilone 1 mg, *n* = 11 (*n* = 5 TKA or THA) Nabilone 2 mg, *n* = 9 (*n* = 6 TKA or THA) 4 total doses: 1-h post induction, then every eight hours with morphine PCA	*n* = 11 (90% female) (2 THA or TKA) Ketoprofen 50 mg *n* = 10 (60% female) (5 THA or TKA) Placebo
Haroutounian et al [26]	Adults with chronic pain > 3 months	*n* = 206 (38% female) (14.6% attrition rate) (*n* = 76 musculoskeletal pain, *n* = 39 low back pain, *n* = 1 avascular necrosis of leg) *n* = 136 Smoked, *n* = 17 oral drops, *n* = 8 drops and smoked, *n* = 9 cookies, *n* = 6 cookies and drops Mean 43.2 g/month added to analgesic regiment THC 6–14% smoked and 11–19% oral CBD 0.2–3.8% smoked and 0.5–5.5% oral	None
Hickernell et al. [9]	Adults post primary THA or TKA	*n* = 81 (65.4% female) (60.5% TKA) Dronabinol 5mg *p.o. B.I.D* with standard pain control during course of hospital stay	*n* = 162 (63.5% female) (57.4% TKA) matched, no placebo given
Holdcroft et al. [25]	Adults with post-operative PCA	*n* = 65 (72% female) (*n* = 23 orthopaedic surgeries) Oral caplet of THC:CBD in 1:0.3 (in 5 mg group) and 1:0.5 ratio given at either 5, 10, and 15 mg of THC	Matched into 3 tiers of dosing, no placebo control
Hunter et al.^a^ [18]	Adults with knee osteoarthritis	*n* = 211 (100% knee osteoarthritis) Synthetic cannabidiol transdermal gel administered for 12 weeks dosed at: 125 mg *B.I.D* (*N* = 106) or 250mg *B.I.D* (*N* = 105)	*n* = 103 placebo gel
Jain et al. [19]	Adults with acute fracture or postoperative pain	*n* = 40 (7.5% female) IM levonantradol given blinded as 1.5, 2, 2.5, or 3 mg doses in addition to meperidine IV, *n* = 10 each dose	*n* = 16 (1.25% female) IM placebo
Levin et al. [20]	Perioperative patients with risk factors for post-operative nausea and vomiting	*n* = 172 (100% female) (*n* = 14 orthopaedic procedures, *n* = 9 spine procedures) 0.5 mg *p.o.* nabilone taken within 3 h prior to induction of anesthesia.	*n* = 168 (100% female) (*n* = 14 orthopaedic procedures, *n* = 9 spine procedures) Identical placebo
Mondello et al. [27]	FBSS patients with moderate to severe pain after spinal cord stimulation therapy	*n* = 11 (45% female) CBD (< 1%)/THC(19%) oleic suspension *p.o.* mean dose 68.5 mg/day	None
Notcutt et al. [22]	Patients with chronic pain recalcitrant to opioids	*n* = 34 (68% female) (0.9% attrition) (*n* = 8 post spine surgery, *n* = 3 post orthopaedic trauma, *n* = 1 arthralgia) Extracts > 95% purity administered sublingually randomized into four groups given for 2 weeks: 2.5 mg THC, 2.5 mg CBD, 2.5 mg CBD, and 2.5 mg CBD, placebo	None
Poli et al. [28]	Adults with chronic pain recalcitrant to two analgesic treatments.	*n* = 725 (62% female) (*n* = 41 arthritis) (53.3% attrition rate over 12 months) 19%THC/< 1%CBD cannabis strain taken as a tea *p.o*.. Dosed at 28 mg/day, equating to 5 mg/day THC. Titrated up with “most” taking 10 mg THC/day	None
Ware et al. [21]	Adults with chronic pain refractory to other treatments	*N* = 215 (49% female) (16.3% “nociceptive pain”) (31.2% attrition rate at 12 months) Herbal cannabis (*n* = 58 smoked, *n* = 130 combination, *n* = 17 oral) 12.5% THC +/-1.5%, median dose 2.5 g/day	*n* = 216 (64.8% female) (18.1% “nociceptive pain”) (15.7% attrition rate at 12 months)
Yassin et al. [23]	Adults with low back pain or sciatica refractory to surgical treatment and/or opioid analgesics	*n* = 46 (52% female) (*n* = 9 post spinal fusion, *n* = 17 spinal stenosis, *n* = 20 disk herniation) (0% attrition) 150–175 mg *Q.I.D.* smoked in addition to tramadol and duloxetine	None
Yassin et al. [24]	Adults with low back pain > 12 months, symptomatic fibromyalgia, and failure of opioid therapy	*n* = 31 (90% female) (*n* = 13 disk herniation, *n* = 21 degenerative changes of spine) (0% attrition) 2/3 g day of smoked cannabis without other analgesics. 1:4 THC:CBD, THC < 5% in addition to oxycodone	None

This table contains the subject population in which the study took place along with characteristics of the cannabinoid assessed and control if applicable

^a^Abstract only

### Control

Six studies had separate control groups. Among RCTs, one study included a positive control of ketoprofen and negative placebo [17], while the remaining three involved only identical placebo as negative control [18–20]. Only two of the non-randomized trials involved a parallel control group: one involved subjects characteristically matched to the intervention group [9] and the other had multiple, statistically significant differences among subjects between intervention and control [21]. Information on control groups is further tabulated in Table 3,

### Analgesic effects

All studies evaluated analgesic effect, using different measures. Most commonly reported were 0–10 point analog scales [17–24, 28], then Brief Pain Index (BPI) [23, 26, 27], McGill pain questionnaire [21], and 0–4 point verbal scales [19, 25]. Eleven found a positive effect [9, 18, 19, 21–28], one found no effect [20], and one a negative effect [17]. RCTs reported mixed efficacy: two demonstrated mitigation of pain [18, 19], one no effect [20], and one increased pain [17]. In the perioperative setting, cannabinoids were found to be efficacious in three studies [9, 19, 25]. Cannabinoids were found effective in all studies evaluating chronic pain of orthopedic etiology [18, 21–24, 26–28]. However, the one RCT in this group found statistical significance only among men [18]. THC or THC in combination with CBD was found to have statistical significance in the reduction of pain vs. placebo, where CBD alone was not statistically significant vs. placebo [22]. In studies that evaluated different dosages of the same cannabinoid, the responses to these differing doses varied across reports. One study reported an exacerbation of pain with increased doses of nabilone (2 mg vs. 1 mg) [17], and another found the pain control provided by 2.5 mg of levonantradol was significantly better than 3 mg [19]. A null effect was also reported with increased dosages, 500 mg/day of topical CBD was not associated with significantly increased analgesia vs. 250 mg/day in the setting of knee osteoarthritis [18]. In contrast, the analgesic effect of 10 mg and 15 mg of oral CBD/THC was found superior to 5 mg in the one dose escalation trial [25].

### Opioid-sparing effects

Seven studies quantified the effect of cannabinoids upon opioid use [9, 23–26], including two RCTs [17, 20]. Five noted a decrease in opioids administered [9, 23–26], while both RCTs reported no change [17, 20]. In the perioperative setting, no change in morphine equivalents given was found by two [17, 20], a reduction of total morphine equivalents given across a hospital stay was found in one [9], and a dose-dependent response in the reduction of rescue analgesia in another [25]. Two studies noted complete cessation of opioid use in the majority of subjects with chronic pain with six [26] to twelve [23] months of cannabinoid use (Table 4).

**Table 4:** Measured effect on pain and opioid use of cannabinoids in identified studies

Study	Effect on pain	Effect on opioid use
Beaulieu [17]	Verbal scale (0–10) placebo, ketoprofen, 1 mg, 2 mg nabilone: Pain at rest: 3.8 ± 0.6, 3.4 ± 0.3, 4.4 ± 0.3, 5.9 ± 0.6*, *p* = 0.0073 Pain with movement: 5.9 ± 0.5, 5.6 ± 0.3, 6.3 ± 0.5, 7.7 ± 0.5*, *p* = 0.0187 *significantly different	mg morphine via PCA placebo, ketoprofen, 1 mg, 2 mg nabilone: 43.3 ± 8.2, 36.9 ± 5.9, 39.0 ± 6.8, 45.4 ± 8.1, *p* = 0.84
Haroutounian et al. [26]	Baseline vs. 6 months: S-TOPS: 83.3 (95% CI 79.2-87.5) to 75.0±4.2 (95% CI 70.8-79.2), *p* < 0.001 BPI Baseline vs. 6 months: Severity 7.5 (95% CI 6.75-7.75) to 6.25 (95% CI 5.75-6.75), *p* < 0.001 Interference: 8.14 (95% CI 7.28-8.43) to 6.71 (95% CI 6.14 to 7.14) *p* < 0.001	Number of subjects on opioid therapy at baseline and at 6 months: 73 to 41, *p* < 0.001 MME/day: 60 (95% CI 45–90) to 45 (95% CI 30–90) *p* = 0.19
Hickernell et al. [9]	Pain via VAS dronabinol vs. control: POD 0: 3.2 ± 2.9, 3.0 ± 3.1, *p* = 0.7 POD 1: 3.0 ± 2.2, 3.3 ± 2.7 *p* = 0.48 POD 2: 3.3 ± 2.8, 3.4 ± 2.8 *p* = 0.85 POD 3: 3.0 ± 2.6, 2.5 ± 2.8 *p* = 0.53	Dronabinol vs. control: Total MME during stay: 252.5 ± 131.5, 313.3 ± 185.4, *p* = 0.088 MME/length of stay: 121.7 ± 76.3, 131.5 ± 111.7, *p* = 0.48
Holdcroft et al [25]	Sum of VRS scores (0–4) from 0.5 to 6 h post-operatively Sum of pain intensity differences at 5, 10, 15 mg: at rest 3,3,5, *p* = 0.01 with movement 0, 1, 1, *p* = 0.7 Total pain relief at 5, 10, 15 mg: 4, 11, 14, *p* = 0.17	Rescue analgesia used in % of subjects in 5, 10, and 15mg groups: 100%, 50%, 25%, *p* < 0.0001 NNT to prevent 1 use of rescue analgesia (relative to 5 mg group) 2.0 (95% CI, 1.5–3.1 with 10 mg, 1.3 (95% CI 1.1–1.7) with 15 mg
Hunter et al.^a^ [18]	Reduction in worst pain (VAS) after 12 weeks of placebo, 250, or 500 mg/day: − 2.37, − 2.64, and − 2.83, no *p* value given. Not statistically significant. Reduction in worst pain after 12 weeks of placebo in men receiving 250 mg/day vs. placebo: − 2.68, − 1.70, *p* = 0.049	Not assessed
Jain et al. [19]	Pain intensity 15 min to 6 h on 4-point scale (0–3), Pain relief on 5 point scale (0–4), Pain analog (0–25) Area under difference curve of pain intensity, relief, and analog: Placebo: 5.20, 9.87 36.32, 1.94 1.5 mg: 9.95*, 16.48*, 71.58**, 3.33* 2.0 mg: 9.84*, 16.90**, 70.33**, 3.30* 2.5 mg:11.26**, 19.10**, 77.61**, 3.50** 3.0 mg 9.10*, 16.08*, 61.18*, 3.90* *p < 0.05, **p < 0.01 (placebo vs. levonantradol).	Not assessed
Levin et al.20	0–10 NRS nabilone vs. placebo, score (SD): NRS 30 min postop (rest) 2.68(3.22), 3.15 (3.41), *p* = 0.47 NRS 30 min postop (movement) 2.75 (3.17), 3.29 (3.42), *p* = 0.70 Maximum pain score (rest) 3.17 (3.15), 3.68 (3.25) *p* = 0.43 Maximum pain score (movement) 3.34 (3.30), 4.0 (3.33) *p* = 0.92	10 mg morphine equivalent (SD) nabilone vs. placebo Intraoperative: 21.3 (15.2) 20.0 (13.4) *p* = 0.40 Post-operative: 5.79 (9.2) 5.35 (6.9) *p* = .62
Mondello et al. [27]	NRS (0–10) baseline vs. 12 months treatment with THC/CBD 8.15 ± 0.98, 4.72 ± 0.9, *p* < 0.0001 BPI baseline vs. 12 months of THC/CBD: General activities 6.72 ± 0.90, 3.63 ± 0.67, *p* < 0.001 Mood 5.54 ± 0.52, 3.63 ± 0.50, *p* < 0.001 Walking abilities 6.45 ± 0.82, 3.27 ± 0.78, *p* < 0.001 Normal work 5.90 ± 0.94, 3.27 ± 0.46, *p* < 0.001 Relations with other people 6.09 ± 1.04, 3.09 ± 0.94, *p* < 0.001 Sleep 6.09 ± 1.44, 3.90 ± 1.30, *p* < 0.01 Enjoyment of life 6.18 ± 0.87, 3.54 ± 1.03, *p* < 0.001	Not assessed
Notcutt et al. [22]	Primary pain location VAS: (median (interquartile range)) placebo 5.9 (2.8–7.3) THC 4.63 (1.74–6.06*, *p* < 0.05 THC:CBD 4.4 (2.6–5.8)*, *p* < 0.01 CBD 5.45 (3.6–7.4) *Significantly different from placebo	Not assessed
Poli et al. [28]	VAS median score at baseline, 1, 3, 6, and 12 months: 9,7,6,5,5 Friedman test, χ^2^ = 61.375 , *p* < 0.001	Not assessed
Ware et al. [21]	VAS average pain intensity 6.78 to 5.54 after 12 months of MCT 5.89 to 6.10 after 12 months in control group McGill Pain Questionnaire total score: 32.29 to 27.50 after 12 months of MCT 26.55 to 26.88 after 12 months in control group	Not assessed
Yassin et al. [23]	VAS baseline vs. 12 months of MCT: Intensity: 9.3 ± 1.3 to 3.3 ± 1.7, *p* < 0.01 Frequency: 8.7 ± 1.4 to 2.0 ± 2.0, *p* < 0.01 BPI at baseline, initiation, 6 months, and 12 months of MCT: Severity: 9.2 ± 1.0, 8.6 ± 1.5, 2.7 ± 1.5, 1.1 ± 0.8, *p* < 0.01 ANOVA Interference: 8.7 ± 1.4, 9.7 ± 1.1, 3.7 ± 1.5, 1.1 ± 0.8, *p* < 0.01 ANOVA	Morphine dose equivalents: 50.7 ± 33.7 prior to MCT 11.1 ± 21.5 after 12 months of MCT *p* < 0.001 *n* = 27 stopped opioid therapy
Yassin et al. [24]	VAS baseline, initiation, 3 months, and 6 months of MCT: 8.1 ± 1.2; 8.1 ± 1.4; 5.3 ± 1.3; 3.3 ± 2.2, *p* < .0001 ANOVA	Opioid consumption significantly decreased

This table includes the method each study assessed pain and quantification of opioid use, as well as data found in each study concerning these variables

^a^Abstract only

### Adverse events

All studies sought to evaluate for AEs, one as the primary outcome [21]. Among six studies with parallel control groups, one found no AEs in the perioperative setting [9] and another no difference in severe AEs over 12 months [21]. Five found significantly greater rates of AEs in their cannabinoid group, including sedation [17, 19], transiently decreased muscular coordination [20], headache and application site xerosis [18], decreased lung function, and upper respiratory complaints [21]. Among all studies, xerostomia [17, 19, 20, 22, 25, 27], headache [18, 20, 21, 27], and drowsiness or sedation [17, 19–22, 25–28] were particularly common. Euphoria or dysphoria occur in three studies [21], and was more common in THC containing preparations vs. CBD or placebo in one study [22]. Three studies [21, 22, 26] sought changes in hematologic markers; there was only one instance of aberration attributed to the intervention: an increase in transaminases [26]. Two studies ended prematurely: one due to a vasovagal event [25] and another due to a paradoxical worsening of pain [17] (Table 5).

**Table 5:** Adverse events recorded in identified studies

Study	Adverse events
Beaulieu [17]	2 mg nabilone: increased sedation and pain Xerostomia, nausea, vomiting, respiratory depression, pruritis common but not different between groups
Haroutounian et al. [26]	Mild-moderate: *n* = 9 (sedation, heaviness, decreased concentration) Severe: *n* = 2 (increased transaminases and acute confusion)
Hickernell et al. [9]	None during length of hospital course (mean 2.3–3 days)
Holdcroft et al. [25]	% of 5,10,15 mg subjects experiencing adverse events: 9%, 30%, 50%, *p* = 0.002 *n* = 4 dizziness, *n* = 4 dysphoria, *n* = 2 xerostomia, *n* = 2 paranoia, *n* = 2 pallor, *n* = 2 tachycardia, *n* = 1 pyrexia, *n* = 1 vomiting, *n* = 1 sensory change, *n* = 1 sleep disturbance, *n* = 1 vasovagal syncope Change in sedation VRS (0–4) at 5, 10, and 15 mg: 0, − 2, − 3, *p* = 0.03
Hunter et al.^a^ [18]	Application site xerosis (3.8 vs. 0.9%) and headache (3.3% vs. 1.9%) were greater than placebo
Jain et al .[19]	2 events with placebo vs. 53 with levonantradol. Most commonly *n* = 19 sedation, *n* = 5 xerostomia, *n* = 4 dizziness, and *n* = 3 weird dreams. No discontinuation, no dose dependent response
Levin et al. [20]	Lack of muscle coordination nabilone vs. placebo: 3/172 vs. 0/168, *p* < 0.0001 No other significant difference in adverse events, other common events: *n* = 31 xerostomia, *n* = 13 headache, *n* = 5 drowsiness
Mondello et al. [27]	*n* = 4 drowsiness, *n* = 3, decreased concentration, *n* = 2 xerostomia, *n* = 2 headache, *n* = 2 nausea/vomiting, *n* = 1 apathy, *n* = 1 puffy lips, *n* = 1 palpitations, *n* = 1 dizziness, *n* = 1 dysmorphic sensation, *n* = 1 mood disorder, *n* = 1 forgetfulness, *n* = 1 urinary retention.
Notcutt et al. [22]	CBD:THC run-in *n* = 21 xerostomia, *n* = 23 drowsiness, *n* = 18 dys/euphoria THC:CBD : *n* = 20 xerostomia, *n* = 14 drowsiness, *n* = 12 dys/euphoria THC: *n* = 17 xerostomia, *n* = 20 drowsiness, *n* = 12 dys/euphoria CBB: *n* = 15 xerostomia, *n* = 8 drowsiness, *n* = 4 dys/euphoria placebo: *n* = 11 xerostomia, *n* = 7 drowsiness, *n* = 1 dys/euphoria *n* = 1 vasovagal syncope with THC
Poli et al. [28]	*n* = 8 confusion, *n* = 6 drowsiness, *n* = 3 worsening pain, *n* = 3 tachycardia, *n* = 3 anxiety, *n* = 3 hallucinations, *n* = 2 pruritis, *n* = 1 depression, *n* = 1 nausea, *n* = 1 increased appetite, *n* = 1 diarrhea, *n* = 1 anal burning, *n* = 1 depression, *n* = 1 dizziness
Ware et al. [21]	Serious events 40 with MCT vs. 56 without MCT at 12 months, IRR = 0.82 (95% CI 0.46–1.46) 1 severe event deemed likely related to cannabinoids: convulsions Total non-serious events 818 with MCT vs. 581 without MCT at 12 months, IRR = 1.64 (95% CI 1.35–1.99) Nervous system disorders, respiratory disorders, infections, psychiatric disorders significantly higher in MCT group Most common: *n* = 41 headache, *n* = 37 nasopharyngitis, *n* = 36 nausea, *n* = 29 somnolence, *n* = 27 dizziness, *n* = 17 vomiting, *n* = 16 cough, *n* = 9 euphoria
Yassin et al. [23]	*n* = 25 increased appetite, *n* = 19 conjunctival injection
Yassin et al. [24]	*n* = 28 conjunctival injection, *n* = 5 increased appetite, *n* = 3 sore throat

## Discussion

The therapeutic application of cannabinoids is an emerging area of research. While Madden et al. focused on study methodology in their reviews appraising the literature through 2017 [8, 29], new studies have since been published [9, 18, 24, 27]. In addition to evaluating these new studies, we sought to narrow the focus of our review to analgesic and opioid-sparing effects of cannabinoids within orthopedic surgery.

The importance of investigation into supplemental analgesics is underscored by the current opioid epidemic in the USA, where nearly 10,000,000 people misused prescription opioids [10] and over 40,000 died of overdose in 2018 [30]. Among all opioid prescriptions, 5.8% [31] to 7.7% [32] were written by orthopedic surgeons, indicating that the field is not immune to the growing opioid problem [33]. While useful for post-operative pain, several studies have demonstrated an alarming trend of use long after convalescence from the index surgery [34–36], even among opioid-naïve patients [37–39]. This trend may be amplified in the future by the projected growth of orthopedic procedures; the number of TKA and THAs are estimated to quadruple from 2010 to 2030 [40]. The neuropharmacology of cannabinoids regarding nociception [41] make them a promising adjunctive analgesic that could potentially mitigate the need for opioids.

The RCTs included in this review demonstrated mixed efficacy regarding pain [17–20], ranging from effective [19] to anti-analgesic [17]. Hunter et al. noted only a significant reduction in pain among men [18]. Notably, Levin et al.’s subjects were entirely female [20] and Beaulieu et al.’s nabilone group was comprised of 85% females [17], while Jain et al. studied > 90% male subjects [19]. Indeed, the sexual dimorphism of cannabinoid pharmacology has been discussed [42, 43] and a recent review noted differing degrees of analgesia among sexes across nine clinical and preclinical studies [43]. All nine non-RCTs demonstrated a positive analgesic effect. However, these studies were non-randomized and only two had parallel control groups limiting the applicability and generalizability of the findings.

The reduction in opioid use was promising; five of seven studies demonstrated reduced opioid consumption with cannabinoid therapy. Holdcroft el al. noted a dose-dependent response in the reduction of rescue analgesia in their trial evaluating post-operative pain [25]. Hickernell et al. reported a reduction in perioperative opioid use; however, this was in the setting of a decreased length of stay in their dronabinol group and no significant change in opioid use per day [9]. A sizeable portion of subjects were able to discontinue opioid therapy at six [26] and twelve [23] months; however, both studies lacked comparative control groups. RCTs evaluating opioid consumption found no difference; however, Levin et al. limited their study to the immediate recovery period within the post anesthesia care unit [20] and Beaulieu et al. limited their study to 24 h [17].

There was a lack of standardization among dose, frequency, concentration, and route of administration. Caplets of synthetic THC were studied in doses of 0.5 mg taken once [20], 5 mg twice a day throughout the hospital course following the index procedure [9], and 1 mg or 2 mg taken four times within 24 h [17]. Levonantradol, another synthetic THC derivative, was administered in 1.5 mg, 2 mg, 2.5 mg, and 3 mg aliquots intramuscularly by Jain et al. [19]. Synthetic transdermal CBD was dosed at either 250 mg or 500 mg a day [18]. Natural cannabinoids ranged in concentrations of < 5% [24] to 95% [22] THC and < 1% [28] to 95% [22] CBD. Oral caplets were dosed 5–15 mg [25], sublingual drops 2.5 mg [22], and oral extractions 28 mg [28] to 68.5 mg [27]. All four studies evaluating smoked cannabis varied in median dose ranging from 600 mg [23] to 2500 mg daily [21].

Cannabinoid choice varied among studies and included synthetic and natural THC, CBD, and THC/CBD combinations. Nottcut et al. demonstrated an increased efficacy of THC and THC/CBD in combination over CBD alone [22]. This finding is also supported elsewhere; a RCT evaluating oncogenic pain found THC/CBD mixtures were more efficacious than placebo and THC alone [44]. The combination of THC and CBD may be useful. Preclinical research has suggested that CBD can mitigate the neuropsychiatric effects produced by THC [45, 46].

Synergism among endogenous cannabinoids [47] has led to the speculation of a similar relationship where THC and CBD may augment the effects of one another when administered synchronously in what has been termed the “entourage effect” [48, 49].

All but one study [9] found AEs. Ware et al. specifically sought to measure AEs primarily with smoked cannabis, noting decreased pulmonary function as well as increased upper respiratory complaints and infections within their cannabis group over the course of 12 months [21]. Smoking was a common route of administration found in this review and many concerns with the combustion of organic plant material exist. Indeed, the deleterious effects of smoked tobacco are well known [50]. Although less studied, cannabis smoke has been associated with negative sequalae such as lung cancer [51] and lower bone mineral densities among heavy users [52].

Among other routes of administration, Hickernell et al. noted no AEs among 81 patients receiving 5 mg dronabinol [9], while studies evaluating nabilone recorded increased rates of impaired muscle coordination [20] and sedation [17] at 0.5 mg and 2 mg respectively. Other common reported effects include nausea, vomiting, altered mentation, and potential drug interactions [4]. Transdermal CBD products are less known in terms of AEs; Hunter et al. found an increased incidence of headache and application site xerosis with this modality vs. placebo [18]. Due to a severe vasovagal event at 15 mg THC/CBD, Holdcroft et al. ceased recruitment in their study. This same study noted a dose-dependent response in AEs from 5 to 15 mg of oral THC/CBD [25]; however, Jain et al. did not demonstrate a dose related response in AEs from 1.5 to 3 mg IM levonantradol [19]. Neuropsychiatric events were rare, consisting mainly of sedation. Euphoria and hallucinations were reported, but rarely. Overall, cannabinoids were well-tolerated within this review; however, this is insufficient evidence to fully evaluate the safety of these compounds. Similarly, the stark differences in route of administration, dose, and actual cannabinoid used underscore the lack of standardization in the use of these compounds and create difficulty in comparing their safety and efficacy across the literature.

Among adverse events, the potential for addiction, chronic dependence, and the resultant socioeconomic effects of employing cannabinoids into orthopedic practice are salient concerns that unfortunately were not assessed within the studies included in this review. However, both the benefits and risks of any therapy must be considered. The ravages of opioid addiction are known, summating in tens of thousands of deaths per year in the USA [30]. The ability of cannabinoids to mitigate this crisis outweighs the negative ramifications in the authors’ opinion. Indeed, in states of the USA where cannabinoid use has become legal, there have been decreased rates of opioid prescriptions, opioid abuse, opioid-associated hospital admissions, and overdose mortality rates [53–55]. Cannabinoids are mechanistically different than opioids resulting in key differences [41]. Opioids depress respiratory drive [56] and indeed this is the primary mechanism of death in acute overdose [57]. Contrarily, no fatal overdoses have been reported with medical or recreational cannabinoid use; furthermore, the quantity of cannabinoids needed to induce a potentially fatal overdose is many multitudes beyond the therapeutic dose [58, 59]. Paradoxically, higher doses of cannabinoids have been reported to cause hyperalgesia [60]. This effect may provide a ceiling to the continual upward titration of dosages. The prolonged use of opioids has been associated with hyperalgesia possibly begetting the need for an ever-increasing dosage to adequately address pain [61]. In contrast, cannabinoids have been shown to not induce hyperalgesia with chronic use [62]. The combination of these factors speaks against chronic dependence upon cannabinoids for pain control.

There are several limitations acknowledged within this review. The dearth of literature existing on cannabinoids in orthopedic surgery left few studies to review. Much of our data was extracted from papers where only a fraction of the subjects underwent orthopedic procedures or had orthopedic conditions. It is impossible to know in these studies what the true impact of cannabinoids was on orthopedic patients given the data of all subjects was combined. The heterogeneity of data and methodology made it impossible to perform a meta-analysis. The overall quality of available data also affects this review, given many studies included are either low to very low in quality and only four RCTs met the inclusion criteria.

## Conclusion

There is sparse data regarding the use of cannabinoids in orthopedic surgery. Only two studies in this review had subjects solely within orthopedics [9, 18]. The applicability of existing RCTs is limited by several factors, chiefly the heterogeneity of intervention and conflicting results. The evidence from non-RCTs demonstrates that cannabinoids may be an effective adjunctive analgesic and possibly curtail opioid usage. However, this is far from convincing, given the overall lack of rigor in their non-randomized design. With exceptions, cannabinoids were well-tolerated within the confines of this review, mainly causing minor AEs. The potential to serve as well tolerated analgesic adjuncts that could mitigate opioid usage make cannabinoids promising agents to investigate. The production of high-quality evidence via well designed RCTs is needed to accurately assess these effects. Attention to route of administration, dosage, choice of cannabinoid, and potential differences in gender response may be important considerations in designing future trials.

### Expectations

It is of the opinion of the authors that cannabinoids may represent an adjunctive solution in providing additional analgesia in an effort to combat the overuse of opioids within orthopedic surgery. Though the current paucity of rigorous evidence makes it difficult to recommend the use of cannabinoids outside of patients involved in research trials. We would expect as legal barriers to studying these compounds continue to dissolve, more research will be performed that will better establish the usefulness of medicinal cannabinoids while better characterizing and refining indications for cannabinoid therapy within orthopedics, dosing, and route of administration. Though impossible to clearly prognosticate, the acceptance of cannabinoids as a legitimate means of pain control could alter prescribing patterns of future orthopedic surgeons and mitigate the current opioid crisis.

## Data Availability

The data used during this current study is available from the corresponding author on reasonable request. All included data were extracted from articles that are cited here-in and are available at their respective sources.

## Abbreviations

PRISMA: Preferred Reporting Items for Systematic Reviews and Meta-Analyses
U.S.: United States of America
FDA: U.S. Food and Drug Administration
THC: Delta-9-tetrahydrocannabinol
CBD: Cannabidiol
AE: Adverse events
GRADE: Grading of Recommendations of Assessment, Development, and Evaluation
RoB 2: Revised Cochrane risk-of-bias tool for randomized trials
RCTs: Randomized controlled trials
ROBINS-I: The Risk of Bias In Non-Randomized Studies–of Interventions
BPI: Brief Pain Index
TLE: Therapeutic level of evidence
M: Moderate
L: Low
VL: Very low
SC: Some concern
PCA: Patient controlled anesthesia
THA: Total hip arthroplasty
TKA: Total knee arthroplasty
ASA: American Society of Anesthesiologist’s scale
B.I.D.: Bis in die
p.o: Per os
IM: Intramuscular
IV: Intravenous
FBBS: Failed back surgery syndrome
Q.I.D.: Quater in die
VAS: Visual analogue scale
MME: Milligram morphine equivalent
VRS: Verbal rating scale
NNT: Numbers needed to treat
S-TOPS: Treatment outcomes in pain survey
POD: Post-operative day
NRS: Numeric rating scale
MCT: Medical cannabis therapy
OR: Odds ratio
CI: Confidence interval
IRR: Incidence rate ratio
